# The complete mitochondrial genome of *Antestiopsis thunbergii* (Gmelin, 1790) (Hemiptera: Pentatomidae) and its phylogenetic implication

**DOI:** 10.1080/23802359.2021.1991242

**Published:** 2021-10-20

**Authors:** Ling Zhao, Jiufeng Wei, Xiaoyun Gao, Chao Chen, Qing Zhao

**Affiliations:** College of Plant Protection, Shanxi Agricultural University, Taigu, China

**Keywords:** *Antestiopsis thunbergii*, pentatomidae, mitochondrial genome, phylogenetic analysis

## Abstract

The complete mitochondrial genome of *Antestiopsis thunbergii* was sequenced and was 15,391 bp long with a base composition of 43.03% A, 9.97% G, 13.04% C and 33.95% T. It contains 37 mitochondrial genes (13 protein-coding genes, 22 transfer RNA genes, 2 ribosomal RNA genes, and a control region). The genome structure, gene order, nucleotide composition, and codon usage of *A. thunbergii* were consistent with those of typical Pentatomidae insects. Phylogenetic analysis implied that *A. thunbergii* belonged to the family Pentatomidae, and each branch had a high Bayesian posterior probability value.

*Antistiopsis thunbergii* (Gmelin, 1790) is one of the most important pests of coffee. It can suck the flowers, buds, leaves, fruits and branches of coffee trees, reducing the yield and quality of coffee beans (Joseph et al. 2019). *Antistiopsis thunbergii* is native to Africa and has now spread to Australia, Myanmar, India, Indonesia, Philippines, Sri Lanka, Thailand, southern China and other places (Rider et al. 2002; Gerry et al. 2016). We sequenced the mitochondrial genome of *A. thunbergii* and determined its phylogenetic position.

Adult specimens of *A. thunbergii* were collected from Credo statio (30°13′30.0″S, 120°41′90.0″E), Western Australia, Australia. The voucher specimens were deposited at the Institute of Entomology, Shanxi Agricultural University (voucher number: BBCRE11 RK37 Msp126; Taigu, China；contact email: zhaoqing@sxau.edu.cn). Whole-genome DNA was extracted from the thoracic muscle of an adult sample using the Genomic DNA Extraction Kit (Sangon Biotech, Shanghai, China). The mitogenome was sequenced on an Illumina MiSeq platform and was assembled using A5-miseq v20150522 software (Coil et al. 2015). Finally, the assembled sequences were annotated using Geneious 8.1.4 software (Kearse et al. 2012) and MITOS (Bernt et al. 2013).

The mitochondrial genome of *A. thunbergii* is a double-stranded closed circular molecule with a total length of 15,951 bp (GenBank accession number: MW679031). It contains 13 protein-coding genes (PCGs), 22 tRNA genes (tRNAs), 2 rRNA genes (*rrnL* and *rrnS*), and a control region. The gene order and orientation of the mitochondrial genes are identical to those of most Pentatomidae species (Yuan et al. 2015). The nucleotide composition of the *A. thunbergii* mitgenome is A 43.03%, G 9.97%, C 13.04% and T 33.95%.

Most PCGs share the start codons of ATN (five with ATG, three with ATT, one with ATA and one with ATC), except that *COI*, *ATP8* and *ND1* starts with TTG. Eight PCGs share the same termination codon of TAA, five PCGs (*COI*, *COII*, *ND3*, *ND4* and *ND6*) are terminated with a single T. The 22 tRNAs range from 52 to 72 bp, and all have a typical cloverleaf secondary structure except *trnI-Ile*, *trnS-Ser ^(AGN)^* and *trnV-Val*, which lack a dihydrouridine arm. The two rRNAs are 1270 bp (*rrnL*) and 818 bp (*rrnS*) long and are separated from each other by *trnV-Val*. The control region is located between the *rrnS* and *trnI-Ile* with a total length of 1285 bp, and has an A + T content of 78.47%.

Phylogenetic analyses of 23 superfamily Pentatomoidea species and two superfamily Lygaeoidea species were conducted using Bayesian inference (BI) methods on the 13 PCGs (Figure 1). In the result, each branch had a high Bayesian posterior probability value. The phylogenetic analysis showed that *A. thunbergii* belonged to the Pentatomidae, which was consistent with morphological analysis. While in the Pentatomidae, the Pentatominae and Phyllocephalinae were mixed together. Among them, *Gonopsis affinis* belonged to Phyllocephalinae, *Eurydema qinlingensis* belonged to Pentatominae, and there was no clear phylogenetic relationship between the four subfamilies. This indicates that more mitochondrial genomes of Pentatomidae need to be determined to better understand the phylogenetic relationship between Pentatomidae subfamilies. In conclusion, the mitochondrial genome data of *A. thunbergii* will help to better understand the molecular evolution and phylogeny.

**Figure 1.**
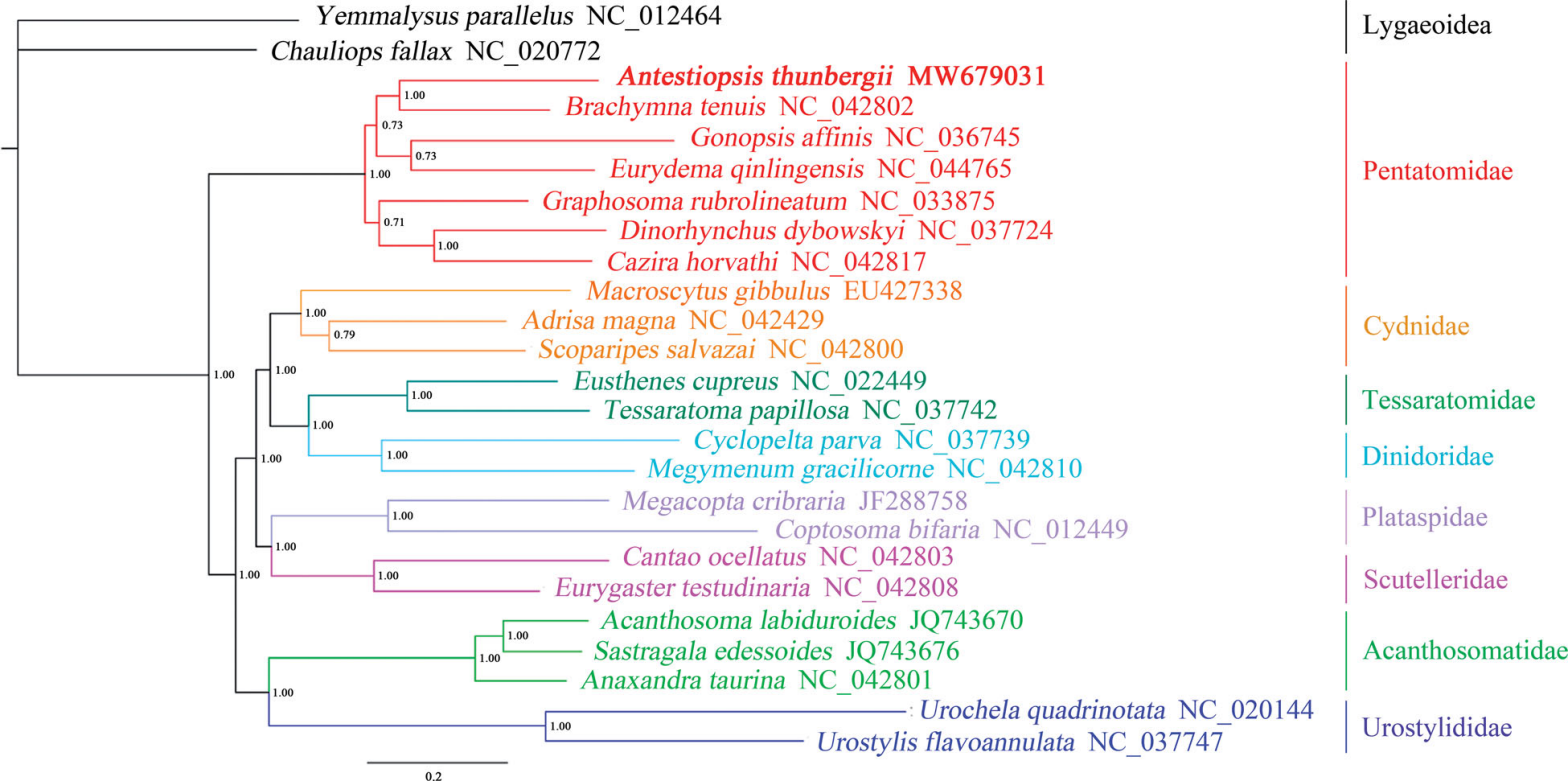
Phylogenetic relationship of *A. thunbergii* within Pentatomoidea inferred from 13 PCGs. Numbers on branches are Bayesian posterior probabilities.

## Data Availability

The genome sequence data that support the findings of this study are openly available in GenBank of NCBI at (https://www.ncbi.nlm.nih.gov/) under the accession no. MW679031. The associated BioProject, SRA, and Bio-Sample numbers are PRJNA765957, SRR16039797, and SAMN21593911, respectively.
